# Multiparametric Monitoring of Early Response to Antiangiogenic Therapy: A Sequential Perfusion CT and PET/CT Study in a Rabbit VX2 Tumor Model

**DOI:** 10.1155/2014/701954

**Published:** 2014-10-15

**Authors:** Jung Im Kim, Hyun-Ju Lee, Young Jae Kim, Kwang Gi Kim, Kyung Won Lee, Jae Ho Lee, Hak Jong Lee, Won Woo Lee

**Affiliations:** ^1^Department of Radiology and Institute of Radiation Medicine, Seoul National University College of Medicine & Clinical Research Institute, Seoul National University Hospital, 101 Daehangno, Jongno-gu, Seoul 110-744, Republic of Korea; ^2^Department of Radiology, Kyung Hee University Hospital at Gangdong, Kyung Hee University College of Medicine, 892 Dongnam-ro, Gangdong-gu, Seoul 134- 727, Republic of Korea; ^3^Biomedical Engineering Branch, Division of Convergence Technology, National Cancer Center, 323 Ilsan-ro, Ilsandong-gu, Goyang-si, Gyeonggi-do 410-769, Republic of Korea; ^4^Department of Radiology, Seoul National University Bundang Hospital, Seoul National University College of Medicine, 82 Gumi-ro, 173 Beon-Gil, Bundang-Gu, Gyeonggi-do 463-707, Republic of Korea; ^5^Department of Internal Medicine, Seoul National University Bundang Hospital, Seoul National University College of Medicine, 82 Gumi-ro, 173 Beon-Gil, Bundang-Gu, Gyeonggi-do 463-707, Republic of Korea; ^6^Department of Nuclear Medicine, Seoul National University Bundang Hospital, Seoul National University College of Medicine, 82 Gumi-ro, 173 Beon-Gil, Bundang-Gu, Gyeonggi-do 463-707, Republic of Korea

## Abstract

*Objectives.* To perform dual analysis of tumor perfusion and glucose metabolism using perfusion CT and FDG-PET/CT for the purpose of monitoring the early response to bevacizumab therapy in rabbit VX2 tumor models and to assess added value of FDG-PET to perfusion CT. *Methods.* Twenty-four VX2 carcinoma tumors implanted in bilateral back muscles of 12 rabbits were evaluated. Serial concurrent perfusion CT and FDG-PET/CT were performed before and 3, 7, and 14 days after bevacizumab therapy (treatment group) or saline infusion (control group). Perfusion CT was analyzed to calculate blood flow (BF), blood volume (BV), and permeability surface area product (PS); FDG-PET was analyzed to calculate SUVmax, SUVmean, total lesion glycolysis (TLG), entropy, and homogeneity. The flow-metabolic ratio (FMR) was also calculated and immunohistochemical analysis of microvessel density (MVD) was performed. *Results.* On day 14, BF and BV in the treatment group were significantly lower than in the control group. There were no significant differences in all FDG-PET-derived parameters between both groups. In the treatment group, FMR prominently decreased after therapy and was positively correlated with MVD. *Conclusions.* In VX2 tumors, FMR could provide further insight into the early antiangiogenic effect reflecting a mismatch in intratumor blood flow and metabolism.

## 1. Introduction

Angiogenesis is an essential component of tumor growth, invasion, and metastasis. Recently, with the continued development of antiangiogenic drugs that target the inhibition of tumor angiogenesis, techniques that can evaluate tumor angiogenesis have also been emphasized in clinical practice. In contrast to other anticancer therapies such as the use of cytotoxic agents and irradiation, after antiangiogenic treatment, a decrease in tumor volume is not an expected treatment response [1]. Rather, quantitative computed tomography (CT) and magnetic resonance (MR) imaging kinetic parameters may be used to obtain insights into underlying tissue pathophysiologic processes of a variety of treatments, allowing prediction of treatment response or monitoring of their effects [2]. For these purposes, perfusion CT has particularly seen increasing use as a method to quantify tumor vascularity and to monitor antiangiogenic response [3–8].

Another modality that has been used to evaluate the angiogenesis of tumors is positron emission tomography (PET). However, published data thus far have shown to be contradictory between different series [9]. Contractor and Aboagye [10] emphasized the requirement of multiphasic effect on FDG (fluorodeoxyglucose) uptake as well as dynamic analytic methods which can overcome the changes in perfusion after antiangiogenic treatment that affects any static imaging protocol. In addition, they suggested that measurement of the pharmacodynamic effects of antiangiogenic therapies with FDG at an earlier time point (within days) may be more appropriate to monitor changes in cell viability.

Another factor that has hampered the assessment of angiogenesis with PET is that standardized uptake values (SUVs) do not thoroughly provide metabolic information of the entire neoplasm although average SUV is partly associated with the tumor volume. Thus, the intratumor heterogeneity with areas of different biological activity would not sufficiently be reflected with SUV values. To compensate for this limitation, in addition to volumetric parameters including metabolic tumor volume (MTV) and total lesion glycolysis (TLG), texture analysis which estimates SUV distribution can be evaluated to characterize the aggressiveness of the tumor and the response assessment of anticancer therapy.

After the introduction of novel oncologic drugs targeted to interfere with specific aberrant biologic pathways involved in tumor development, the limitations of current structural imaging approaches have been further highlighted. Although the application of functional imaging such as FDG-PET and tumor perfusion imaging as markers of tumor response has been increasing in both research and clinical settings, these functional imaging techniques have been used in isolation [11]. There have been few studies in which both sequential perfusion and FDG uptake have been measured after antiangiogenic treatment.

Therefore, the purpose of this study was to undertake dual analysis of tumor perfusion and glucose metabolism after initiation of bevacizumab, a monoclonal antibody against the vascular endothelial growth factor, using perfusion CT and FDG-PET/CT in a VX2 tumor model in rabbits and to assess the possible added value of variable FDG-PET-derived indices to perfusion CT in monitoring early treatment response.

## 2. Materials and Methods

### 2.1. Study Design

This study was approved by the animal care committee of our institute. The rationale for selecting VX2 tumors for our experimental model is as follows: its blood supply is similar to that of human hepatocellular carcinomas, with rapid growth of the tumor, allowing it to grow relatively large and easily identified on imaging [12–14]. Twelve adult New Zealand white rabbits weighing 3.0 to 3.5 kg were used. For each rabbit, a 24-gauge medicut was inserted into an auricular vein, and anesthesia was induced with intravenous ketamine hydrochloride (50 mg per kg of body weight; Ketamine, Yuhan, Korea) and 2% xylazine (0.1 mL/kg; Rompun, Bayer, Germany). Thereafter, the bilateral back parallel to the spine of the rabbits was shaved and sterilized, and the rabbits were put in the prone position on the table. Under ultrasound (US) guidance, a 21-gauge Chiba needle was inserted into the bilateral back muscle at the level of the kidney. Then, a VX2 tumor suspension (0.1 mL) was slowly injected into each back muscle through the Chiba needle.

On one week after tumor implantation when the tumors were expected to be round in shape with a diameter of approximately 1 cm, a baseline perfusion CT as well as PET/CT was performed for each rabbits. The rabbits were randomly allocated (6 per group) to receive serial injections of either bevacizumab (10 mg/kg; Avastin, Genentech) or the same dose of normal saline (treatment group and control group, resp.). Follow-up perfusion CT and PET/CT scans were acquired 3, 7, and 14 days after the baseline study. Each PET/CT scan was performed within 6 hours after perfusion CT scanning.

### 2.2. Perfusion CT and FDG-PET/CT Imaging

During CT imaging and PET/CT, animals were sedated with an intramuscular injection of 5 mg/kg of Xylazine hydrochloride (Rompun; Bayer Korea, Seoul, Korea) and 15 mg/kg of Zoletil mixture (Zoletil 50, Virbac Lab., Carros Cedex, France).

#### 2.2.1. Perfusion CT Imaging

The rabbits were fixed on a CT board in the supine position by applying a thoracoabdominal bandage to reduce motion artifacts. The whole perfusion CT imaging was performed with a 64-detector row dual-source scanner (Siemens Definition; Siemens Medical Solutions, Erlangen, Germany). Noncontrast CT (80 kV; 120 mAs; detector configuration 20 × 0.6 mm; rotation time 1 second; section thickness 5.0 mm; reconstruction interval 5.0 mm; and reconstruction kernel H31s) was performed to confirm the complete coverage of the tumor. Marking of the tumor injection site provided a good guide for tumor localization. An experienced radiologist (J.I.K.) with seven years of experience in chest perfusion CT analyzed the unenhanced CT images and selected a fixed scanning range to cover the entire mass with care taken to avoid the possible exclusion of the peripheral portion of the mass during free breathing.

A total of 6.5 mL of a nonionic iodinated contrast medium (Ultravist 370, Schering, Berlin, Germany) was injected using a dual-phase injection protocol (3 mL at 0.5 mL/sec and 3.5 mL at 0.1 mL/sec followed by a 1.5 mL saline flush at 0.1 mL/sec) and a dual-head power injector (MedradStellant Dual; Medrad, Indianola, Pa). Perfusion CT scanning was started 2 seconds after injection with free breathing dynamic acquisition. The CT parameters were as follows: 120 kV, 150 mAs; detector configuration 24 × 1.2 mm; rotation time 0.33 seconds; section thickness 3.0 mm; reconstruction interval 2 mm; and reconstruction kernel B20f. Dynamic CT scans were obtained 40 times for each mass with a 1.5 second interval for the first 20 scans, a 3 second interval for the next 10 scans, and a 6 second interval for the remaining 10 scans; the total scanning time was 120 seconds.

#### 2.2.2. PET/CT Imaging

All rabbits were fasted for at least 6 hours prior to intravenous injection of 37 MBq (1 mCi) of FDG. Before scanning, urinary voiding was performed with a 6-F urinary catheter. Scanning was performed 45 minutes after the injection of FDG using a dual modality PET/CT scanner (Gemini; Philips Medical Systems, Milpitas). CT was performed for attenuation correction, and an emission scan was performed in three-dimensional mode with a 256 × 256 matrix. PET images were reconstructed using an ordered-set expectation-maximization algorithm and CT scans were reconstructed with a section thickness of 4 mm to match the parameters of the PET scan.

### 2.3. Perfusion CT Data Processing

A radiologist (J.I.K., who had 8 years of experience in interpreting perfusion images) measured all perfusion parameters using commercially available software (Syngo VPCT, Siemens Healthcare, Forchheim, Germany). The measurement was performed with computerized motion correction using whole tumor coverage. Arterial input was measured by placing a circular region of interest (ROI) at the level of renal artery os within the abdominal aorta and an arterial time-enhancement curve was automatically generated with the software. A volume of interest (VOI) of the whole tumor was manually drawn around the tumor outline in all three planes (axial, coronal, and sagittal) with exclusion of the adjacent muscle. The following settings were adopted for segmentation: tissue upper and lower limits of 150 and −50 HU, respectively, reference vessel input window width and a center of 300 and 150 HU, respectively, and percentage of relative threshold inside and outside of 50 and 50, respectively.

At each measurement of perfusion CT, blood flow (BF; mL/100 mL/min), blood volume (BV; mL/100 mL), and permeability surface area product (PS; mL/100 mL/min) were obtained. With the software (Syngo VPCT, Siemens Healthcare, Forchheim, Germany), blood flow was calculated using the maximum slope method [15] and blood volume and PS were calculated using Patlak analysis [16].

### 2.4. PET/CT Analysis

**Figure 1:**
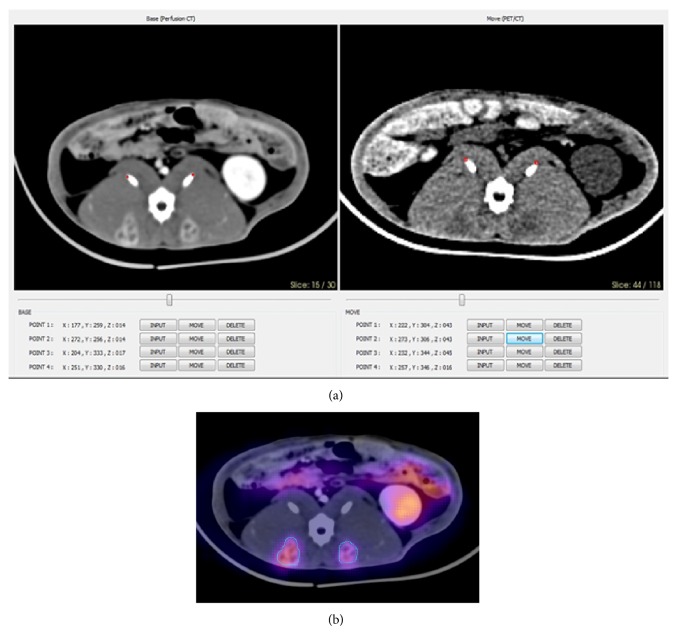
Coregistration of perfusion CT and PET images on day 7: rabbit number 3 in the control group. (a) A contrast-enhanced CT image from the thirty-first frame (time point) of perfusion CT and noncontrast CT image from PET/CT acquisition were coregistered to the same locations. (b) PET and coregistered perfusion CT images were rigidly fused using in-house developed software and ROIs were manually delineated throughout the whole tumor at each axial image.

The CT scan of the PET/CT acquisition and the thirty-first frame (time point) of the perfusion CT scan were coregistered to the same locations. Thereafter, the PET scan and coregistered perfusion CT scan were rigidly fused using in-house developed software, which uses the three-dimensional registration and color mapping hardware (Figure 1). PET and perfusion CT data were initially transferred to the software in DICOM format.

For each tumor, manually delineated ROIs were placed along the boundary of the tumor on axial fused images 3 mm in section thickness, in which the tumor was easily demarcated with guidance from coregistered perfusion CT images. Our in-house software calculated the maximum standardized uptake value (SUVmax), mean SUV (SUVmean), tumor volume (TV), and total lesion glycolysis (TLG; TV × SUVmean).

All textural parameters were calculated from the manually delineated ROI as described above. Voxel values were first resampled to yield a finite range of 128 discrete values between the minimum and maximum SUV in the tumor, using

(1) $$\begin{array}{c} R\left(x\right)=128*\left(\frac{I\left(x\right)-\text{SUVmin}}{\text{SUVmax}-\text{SUVmin}+1}\right), \end{array}$$

where *I*(*x*) is the SUV of voxel *x* in the original image, SUVmin and SUVmax are the minimum and maximum SUV in the VOI, and *R*(*x*) is the resampled value of voxel *x* [17].

Two 3D matrices depicting texture heterogeneity were estimated from the delineated tumors. The grey level cooccurrence matrix (GLCM), describing the pair-wise arrangement of voxels is a 3D matrix related to texture heterogeneity at a local level [18].

In this study, we focused on 2 textural indices that were initially calculated: homogeneity and entropy calculated from the GLCM as defined by Haralick et al. [18], Amadasun and King [19], and Txier et al. [20]. While homogeneity measures the homogeneity of a pixel pair, entropy measures the randomness of gray-level distribution.

The flow-metabolic ratio (FMR, the ratio of contrast agent delivery to tumor glucose metabolism) was also determined for each patient by dividing blood flow by SUVmax [21]. The FMR, a combined assessment of intratumor blood flow and glucose metabolism using FDG-PET and perfusion CT, has been recently introduced as a promising parameter in clinical practice with the potential to risk-stratify patient.

### 2.5. Histopathologic Evaluation

After completion of serial perfusion CT scanning, all rabbits were sacrificed with an intravenous injection of a lethal amount of sodium thiopental (Pentothal) and were frozen in a refrigerator at −70°C in the same position used for CT imaging. More than 24 hours later, the rabbits were cut in the axial plane according to the guidance of the transverse line, 5 mm in thickness, marked on the skin. After each slice of the specimens was fixed with 10% phosphate-buffered formaldehyde and embedded in paraffin, pathologic specimens (approximately 5 *μ*m thick) were obtained. For microvessel density (MVD) measurement, these pathologic slides were immunohistochemically stained specifically for the endothelial antigen CD31, which is traditionally used to evaluate tumor angiogenesis. Counterstaining with hematoxylin-eosin was performed for regular histologic characterization of the tumor.

MVD was quantified by counting the number of vessels plus immunoreactive endometrial cells per 200x high power field in four vascular “hot spots” within the malignant tumor with the mean number reported as the final MVD [22]. Hot spots were selected at low magnification (40x) and corresponded to areas that showed stronger CD31 staining and, consequently, higher vascular density than the rest of the tissue. MVD was calculated when any endothelial cell or cell cluster showed CD31 staining and was clearly separate from adjacent tissue elements [23–25]. ScanScope CS (Aperio Technologies, Inc., Vista, CA, USA) was used to scan the entire tissue sections at ×40 magnification and Aperio ImageScope v11.0.2.782 (Aperio Technologies, Inc., Vista, CA, USA) was used for quantification of MVD.

### 2.6. Statistical Analysis

Within the framework of the mixed-effect model, a repeated-measure analysis was used to compare the mean change of measure parameters from baseline. In addition, measured data between the treatment and control groups were compared at each time points. Rabbit and tumor identification were both used as random effects to accommodate possible correlations between tumors from the same rabbit and measurements with the same rabbit across time.

To assess the relationship between perfusion and metabolic parameters as well as between measured parameters and CD 31 expression, Pearson's or Spearman's correlation coefficients were applied under the linear mixed effect model. All statistical analyses were performed using the *R* statistics package (v.3.1.0; http://www.R-project.org/) and SAS 9.2 software (SAS Institute); a two-tailed *P* value < 0.05 was considered to indicate a statistically significant difference for all statistical analyses.

## 3. Results

In two rabbits, perfusion CT data were incomplete: perfusion CT on day 3 of one rabbit in the control group and baseline perfusion CT of another rabbit in the treatment group were not suitable for imaging registration owing to movement during CT scanning. A baseline FDG-PET/CT data of one rabbit in the control group was also not suitable for imaging registration due to motion artifacts. In addition, two rabbits each in the control and treatment groups unexpectedly expired after imaging on day 7.

**Figure 2:**
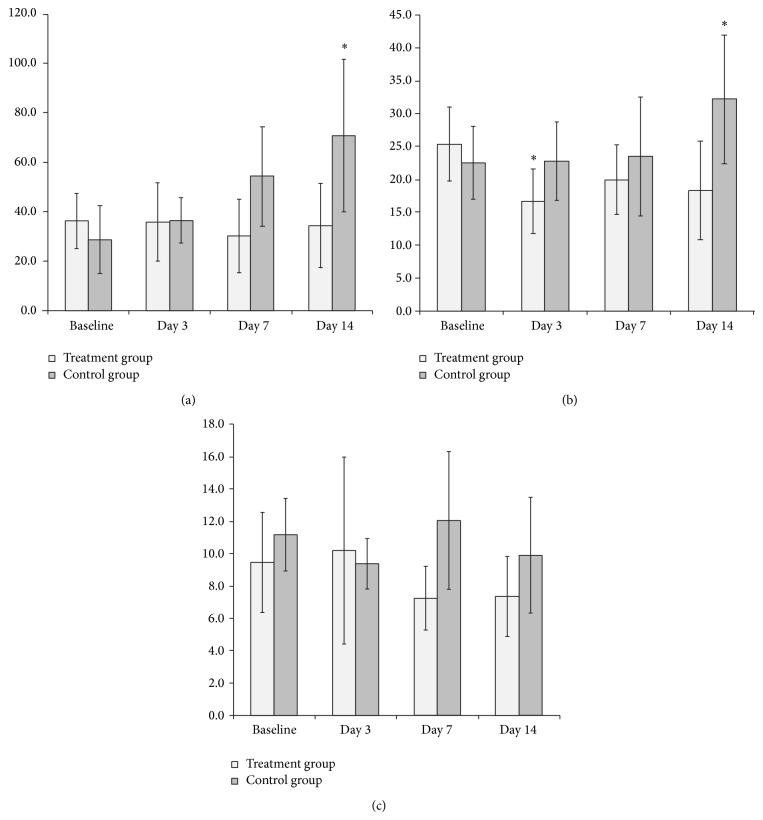
Averaged (a) BF, (b) BV, and (c) PS before and at different time points after bevacizumab therapy. In the control group, BF increased gradually and both BF and BV on day 14 demonstrated significant increases. In the treatment group, a significant decrease in BV on day 3 was observed. ∗ is significant change compared to the baseline, BF is blood flow, BV is blood volume, and PS is permeability surface area product.

Both in the control and the treatment groups, the volume of tumors significantly increased during follow-up with no statistically significant differences between the two groups (Figure 2 and Table 1).

### 3.1. Sequential Assessment of Perfusion CT and FDG-PET/CT

#### 3.1.1. Perfusion CT

Changes in the mean values of BF, BV, and PS over time with averaging of the parameters of the entire tumor in each group are summarized in Figure 2 and in Table 1. In the control group, BF and BV showed a trend toward an increase over time. A significant increase in BF and BV was observed on day 14 (*P* < 0.05, both), compared with the same parameters at baseline (Figure 2). In the treatment group, a significant decrease in BV was observed on day 3 and day 14 (*P* < 0.05), compared with baseline, in contrary to BF in which changes from baseline were unnoticeable (Figure 2). Changes in the mean values of PS were minimal over time both in the control and the treatment groups (Figure 2).

#### 3.1.2. FDG-PET/CT

**Figure 3:**
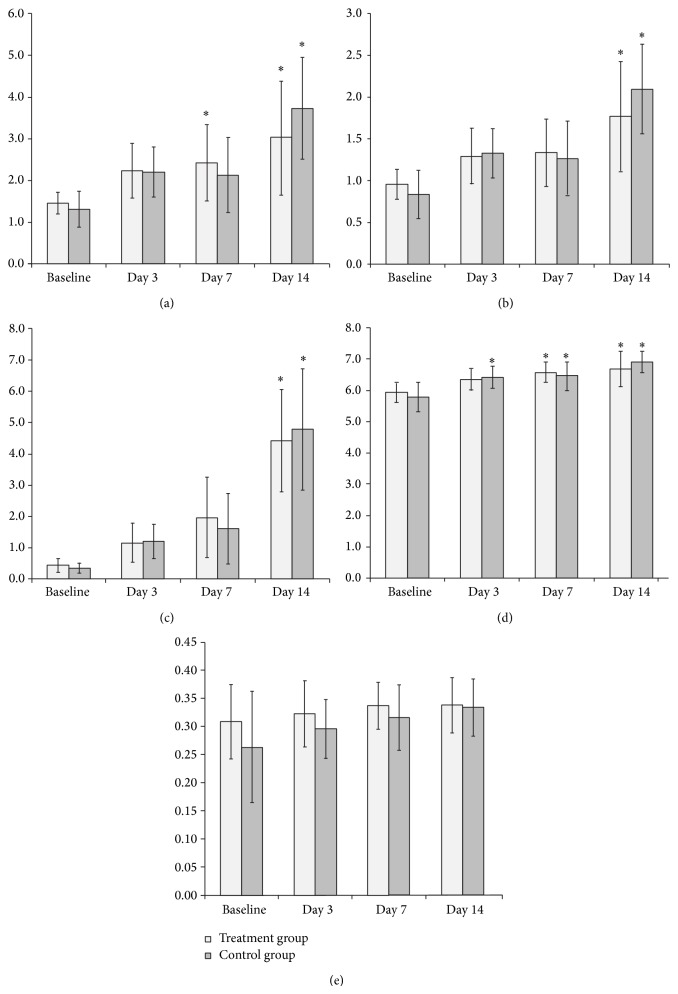
Averaged (a) SUVmax, (b) SUVmean, (c) TLG, (d) entropy, and (e) homogeneity before and at different time points after bevacizumab therapy. SUVmax, SUVmean, TLG, and entropy showed an increasing trend in both groups. ∗ is significant change compared to the baseline, SUV is standardized uptake value, TLG is total lesion glycolysis, and FMR is flow-metabolic ratio.

Changes in the mean values of SUVmax, SUVmean, TLG, local entropy, local homogeneity, and FMR over time with averaging of the parameters of the entire tumor in each group are shown in Figure 3 and in Table 1. All parameters obtained from FDG-PET/CT except homogeneity showed a significant increase over time in both groups (*P* < 0.01 ~ 0.001) (Figure 3). Particularly on day 14, a marked increase in SUVmax, SUVmean, TLG, and entropy was observed in both groups (*P* < 0.001). In contrast, changes in local homogeneity were minimal over time in both groups (Figure 3).

FMR showed a significant decrease over time in the treatment group but no significant difference over time in the control group (Figure 3). In the treatment group, a significant increase in FMR was observed on days 7 and 14 (*P* < 0.05, both), compared with baseline.

### 3.2. Treatment versus Control Group: Perfusion, Metabolism, and MVD

**Figure 4:**
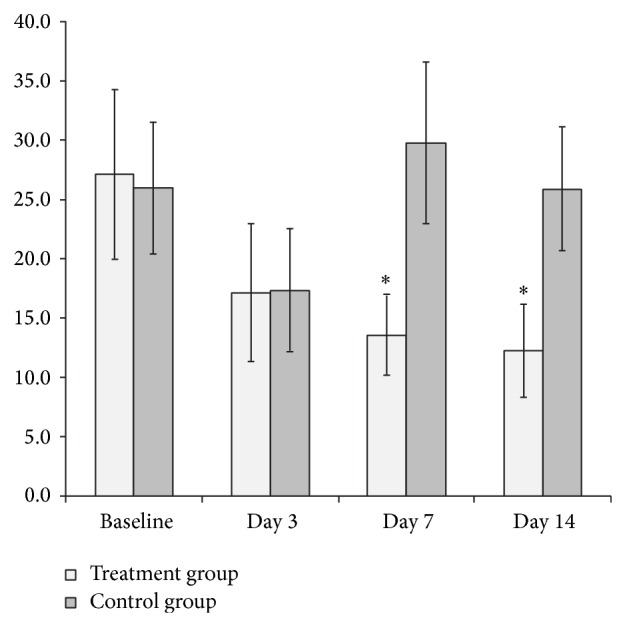
Averaged flow-metabolic ratio (FMR) before and at different time points after bevacizumab therapy. FMR showed a distinct decreasing trend in the treatment group whereas no significant change was identified in the control group. In particular, FMR on day 7 and day 14 significantly decreased compared with baseline.

BF on day 7 of the treatment group was significantly lower than in the control group (*P* < 0.05), and the difference measured on day 14 was still significant between the two groups (*P* < 0.05) (Table 1). In addition, BV on day 3 of the treatment group was significantly lower than in the control group (*P* < 0.05), and a further significant difference was measured on day 14 (*P* < 0.01) (Table 1). None of the PET-derived parameters showed statistically significant differences between the two groups (Table 1). However, FMRs on days 7 and 14 of the treatment group were significantly lower than in the control group (*P* < 0.05) (Figure 4).

**Figure 5:**
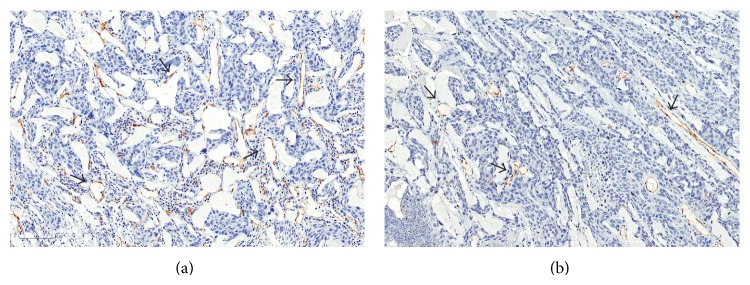
Representative microvessel density (MVD) of (a) control and (b) treatment groups: immunostaining with CD31 antibody highlights vessels (arrows). Intratumoral MVD of the right tumor of the rabbit number 5 (control group) is 26.25 and the left tumor of the rabbit number 2 is 9.25 (original magnification, ×200).

The mean MVD of the treatment group was significantly lower than the control group (8.75 ± 2.49 (range, 4~12) versus 21.50 ± 3.49 (range, 17~26), resp.; *P* < 0.001) (Figure 5).

### 3.3. Correlation Analysis

Both in the treatment and in the control groups, no significant correlation was demonstrated between blood flow and metabolic parameters. However, in the treatment group, BF and FMR correlated significantly with MVD (*r* = 0.709, *P* < 0.05; *r* = 0.782, *P* < 0.01, resp.). In the control group, only BF correlated significantly with MVD (*r* = 0.733, *P* < 0.05).

## 4. Discussion

Antiangiogenic agents have become an essential components of cancer research, and among them, bevacizumab (Avastin; Genentec) has been the most widely used antiangiogenic agent. The development and validation of biomarkers for the prediction of response to antiangiogenic therapy is an area of increasing importance, particularly given recent concerns surrounding the effectiveness of such therapies in prolonging patient survival as well as their potential effects on metastasis [26]. Perfusion CT, an advanced functional imaging biomarker, cannot provide only regional morphologic maps but also quantitative measurements of various tissue hemodynamic parameters [3]. Several previous studies have already reported the predictive values of perfusion CT in antiangiogenic therapy, considering that changes in tumor vascularity precede the changes in tumor size within days of initiating antiangiogenic therapy [3, 7, 27–29]. However, the usefulness of FDG-PET in detecting the response to antivascular endothelial growth factor (VEGF) therapies has been limited [30]. Our study evaluated the early therapeutic response to bevacizumab using serial FDG-PET/CT and perfusion CT and assessed the possible additional value of FDG-PET to perfusion CT.

We found that in the treatment group, there were no significant changes in BF and PS other than a weak declining trend over time in BV. This may have resulted from intratumor vascular normalization of early antiangiogenic effects on perfusion parameters, previously mentioned by García-Figueiras et al. [31], although BF did not show increase in our study. However, in the control group, BF and BV showed a significant increase over time implying an increase in intratumor mature vascular density [32].

To the contrary, there were no significant differences in SUVmax and SUVmean between the two groups even though the values of the treatment group were slightly lower than in the control group. As the tumors became larger, SUVmax and SUVmean became significantly higher regardless of antiangiogenic treatment. This is comparable to the results of Miles et al.'s study [33] in which greater FDG uptake of non-small cell lung cancer (NSCLC) was linked to larger tumors and to Willett et al.'s study [4] using perfusion CT and FDG-PET reporting significant falls in perfusion but no change in glucose metabolism after bevacizumab treatment.

Recently, metabolic-volumetric variables and texture analysis have been introduced as new emerging parameters for PET/CT therapy monitoring. As SUVmax reflects the metabolic activity of the most aggressive cells and lacks prognostic significance, TLG has been suggested as a quantitative parameter for FDG-PET [34]. Indeed, a few works showed that TLG can be significant prognostic factors in various tumors [35]. However, in this study, although the tumor volume (TV) was meticulously acquired using manual delineation on coregistered perfusion CT and PET images, we could not find any statistically significant differences in TLG between the treatment and control groups. Similar with SUVmax and SUVmean, the TLG of both groups demonstrated significant sequential increase after initiation of bevacizumab. Considering that bevacizumab is not only a cytostatic agent but that SUVmax or SUVmean are dependent on tumor size, no significant differences in tumor volume and TLG between the treatment and control groups may be an appropriate result.

In addition to quantitative analysis of FDG-PET, characterization of tumor FDG distribution has been introduced as a useful resource in predicting therapeutic response. Intratumor FDG activity distribution may be assessed in a global, regional, or local fashion, allowing the assessment of corresponding global, regional, or local patterns of biologic heterogeneity [20]. Among the variable texture parameters, the valuation of local homogeneity and entropy may provide the best outcomes for characterization of local nonuniformities. In this study, however, changes in local homogeneity of the treatment group over time were minimal in both groups and the difference between the two groups was also diminutive. On the other hand, local entropy showed an increasing trend over time in both groups although the difference between the two groups was also minimal, consistent with the results of SUV and TLG.

One of our more interesting observations was that FMR had significantly decreased since bevacizumab initiation despite the fact that there was a considerable increase in SUVmax with no significant change in BF over time; FMRs on day 7 and day 14 were significantly lower, compared with baseline values. Similar observation has been reported for the antivascular agent endostatin on various advanced cancers where high doses of endostatin resulted in decreased tumor perfusion but increased glucose metabolism [36]. Furthermore, in the control group, FMR did not demonstrate a noticeable change over time. This suggests that there may be a mismatch of flow and metabolism following antiangiogenic therapy, possibly indicating drug-induced hypoxia and subsequent stimulation of glucose metabolism. Although a significant decrease in FMR and BV was observed after bevacizumab therapy, FMR showed considerably higher changes and a greater tendency to decrease compared to the changes in BV. We were also able to identify a significant positive correlation between FMR and MVD in the treatment group. Given that MVD reflects tumor angiogenesis, the degree of change in FMR may potentially indicate the degree of the antiangiogenic effect. Ultimately, FMR may provide further biologic insights into early antiangiogenic effects reflecting the mismatch between intratumor blood flow and metabolism.

Our results support the view of Miles et al.'s study in which they posit that imaging tumor blood flow and metabolism has potential applications for the noninvasive characterization of tumor aggression, allowing novel subclassification of response with opportunities for personalized cancer care [11].

There are still controversial results concerning the relationship between perfusion and glucose metabolism in the literature that can be classified as showing positive, showing negative, and showing no correlation [37]. In our study, we found no correlation between the perfusion and metabolic parameters nor among metabolic-volumetric and texture parameters. Furthermore, we also found no correlation between MVD and FDG uptake-derived parameters.

Our study has several limitations. First, subcutaneous tumor models may not accurately reflect that of real patients in the clinical setting. Secondly, our study included only a small number of tumors, and thus further studies with larger groups of patients are warranted to support our results.

## 5. Conclusions

The predictive value of FDG-PET was shown to be limited in monitoring the early effect of bevacizumab despite applying texture analysis and volumetric assessment. However, we found that FMR showed a significant sequential decrease over time after bevacizumab therapy which positively correlated with MVD. Thus, our study proposes a potential role of FMR in monitoring the early response to antiangiogenic therapy which will need to be confirmed from larger clinical studies in the near future.

## Figures and Tables

**Table 1:** Perfusion and metabolic parameters between the treatment and the control groups at each time point.

Parameters	Before		3 D		7 D		14 D	
	Treatment	Control	Treatment	Control	Treatment	Control	Treatment	Control
Tumor volume (cm^3^)	0.18 ± 0.08	0.18 ± 0.10	0.53 ± 0.32	0.43 ± 0.23	0.79 ± 0.47	0.76 ± 0.57	1.53 ± 1.11	1.88 ± 1.32
Blood flow (mL/100 mL/min)	36.16 ± 11.15	28.64 ± 13.78	35.82 ± 25.93	36.44 ± 9.22	**30.05** ± **14.87**	**54.13** ± **30.11**	**34.34** ± **17.10**	**70.75** ± **50.65**
Blood volume (mL/100 mL)	25.36 ± 5.62	22.56 ± 5.56	**16.71** ± **4.91**	**22.81** ± **5.95**	19.99 ± 5.30	23.54 ± 9.04	**18.35** ± **7.48**	**32.20** ± **9.76**
PS (mL/100 mL/min)	9.47 ± 3.09	11.18 ± 2.24	10.18 ± 5.81	9.39 ± 1.58	7.246 ± 1.97	12.06 ± 7.25	7.36 ± 2.48	9.91 ± 3.59
SUVmax	1.45 ± 0.26	1.31 ± 0.43	2.23 ± 0.65	2.21 ± 0.60	2.42 ± 0.92	2.13 ± 0.89	3.03 ± 1.36	3.73 ± 1.22
SUVmean	0.96 ± 0.18	0.84 ± 0.29	1.30 ± 0.33	1.33 ± 0.29	1.33 ± 0.40	1.27 ± 0.45	1.77 ± 0.66	2.10 ± 0.54
TLG (cm^3^)	0.43 ± 0.22	0.35 ± 0.16	1.16 ± 0.63	1.20 ± 0.55	1.96 ± 1.28	1.61 ± 1.13	4.43 ± 3.63	4.78 ± 3.95
Entropy	5.95 ± 0.32	5.80 ± 0.47	6.36 ± 0.34	6.41 ± 0.35	6.58 ± 0.32	6.46 ± 0.46	6.69 ± 0.57	6.91 ± 0.34
Homogeneity	0.31 ± 0.07	0.26 ± 0.10	0.32 ± 0.06	0.30 ± 0.05	0.34 ± 0.04	0.32 ± 0.06	0.34 ± 0.05	0.33 ± 0.05
FMR	27.10 ± 12.19	25.19 ± 20.54	17.12 ± 12.80	17.33 ± 7.20	**13.55** ± **6.41**	**29.75** ± **22.78**	**12.24** ± **6.92**	**25.87** ± **29.22**

Data are presented as mean ± standard deviation.

Boldface indicates significant difference between the treatment and the control groups.

PS: permeability surface area product.

SUV: standardized uptake value.

TLG: total lesion glycolysis.

FMR: flow-metabolic ratio.
